# Transcriptome Analysis Reveals Key Cold-Stress-Responsive Genes in Winter Rapeseed (*Brassica rapa* L.)

**DOI:** 10.3390/ijms20051071

**Published:** 2019-03-01

**Authors:** Li Ma, Jeffrey A. Coulter, Lijun Liu, Yuhong Zhao, Yu Chang, Yuanyuan Pu, Xiucun Zeng, Yaozhao Xu, Junyan Wu, Yan Fang, Jing Bai, Wancang Sun

**Affiliations:** 1 College of Agronomy, Gansu Agricultural University, Lanzhou 730070, China; 18189560623@163.com (L.M.); 18894310220@163.com (Y.Z.); changy157@163.com (Y.C.); vampirepyy@126.com (Y.P.); xuyaozhao@126.com (Y.X.); wujuny@gsau.edu.cn (J.W.); ffyv@163.com (Y.F.); bj741912523@163.com (J.B.); 2 Gansu Provincial Key Laboratory of Aridland Crop Science, Lanzhou 730070, China; liulj198910@163.com; 3 Department of Agronomy and Plant Genetics, University of Minnesota, St. Paul, MN 55108, USA; jeffcoulter@umn.edu; 4 College of Agronomy and Biotechnology, Hexi University, Zhangye 734000, China; xiucunzeng@126.com

**Keywords:** *Brassica rapa* L., cold tolerance, differentially expressed genes, peroxisome biogenesis

## Abstract

Low ambient air temperature limits the growth and selection of crops in cold regions, and cold tolerance is a survival strategy for overwintering plants in cold winters. Studies of differences in transcriptional levels of winter rapeseed (*Brassica rapa* L.) under cold stress can improve our understanding of transcript-mediated cold stress responses. In this study, two winter rapeseed varieties, Longyou-7 (cold-tolerant) and Lenox (cold-sensitive), were used to reveal morphological, physiological, and transcriptome levels after 24 h of cold stress, and 24 h at room temperature, to identify the mechanism of tolerance to cold stress. Compared to Lenox, Longyou-7 has a shorter growth period and greater belowground mass, and exhibits stronger physiological activity after cold stress. Subsequently, more complete genomic annotation was obtained by sequencing. A total of 10,251 and 10,972 differentially expressed genes (DEG) were identified in Longyou-7 and Lenox, respectively. Six terms closely related to cold stress were found by the Gene Ontology (GO) function annotation. Some of these terms had greater upregulated expression in Longyou-7, and the expression of these genes was verified by qRT-PCR. Most of these DEGs are involved in phenylpropanoid biosynthesis, plant hormone signal transduction, ribosome biogenesis, MAPK signaling pathway, basal transcription factors, and photosynthesis. Analysis of the genes involved in the peroxisome pathway revealed that Longyou-7 and Lenox may have different metabolic patterns. Some transcription factors may play an important role in winter rapeseed tolerance to cold stress, and Longyou-7 is slightly slower than Lenox. Our results provide a transcriptome database and candidate genes for further study of winter rapeseed cold stress.

## 1. Introduction

Low air temperature is one of the key environmental factors to cope with in the process of crop production in northwestern China, as it limits crop species, growth, yield, and quality in [1]. Cold tolerance is a necessary trait for crops to withstand low temperatures, especially for overwintering crops [2]. Cold tolerance has been studied in several plant species, including *Arabidopsis thalina*, *Triticum aestivum*, *Jatropha curcas* L., and *Lilium lancifolium* [3,4,5,6]. More than 1000 cold- and drought-stress-regulated genes have been detected in the *Arabidopsis* response to cold stress. Overexpression of *CbADH1* in *Arabidopsis thaliana* improved cold tolerance, as indicated by a decreased lethal temperature [7]. Cold stress response is also regulated by epigenetic changes. DNA methylation changes during winter dormancy in *Malus domestica* [8], and in the response to cold stress of chickpeas [9]. MYBS3-mediated cold signaling, as a key factor in cold adaptation of banana, and heterologous overexpression of *MpMYBS3* in banana has shown that transgenic lines had greater cold tolerance than the wild type [10]. Cold treatment initiates the expression and activity of sugars and amino acids in plants, which are essential for the synthesis of functional proteins and serve as precursors to a variety of metabolites with functions for cold tolerance [11]. However, the complex molecular mechanisms involving physiological processes associated with cold acclimation have not been completely elucidated.

Solexa/Illumina RNA sequencing (RNA-seq) has recently been used to identify cold tolerance genes in different species [12,13]. Studies investigating plant responses to cold stress have involved transcriptome profiling for many plant species [14], including *Arabidopsis thaliana* [12,15], *Brassica napus* [16], *Camellia sinensis* [17], and many others [5,13,18,19,20]. RNA-Seq analysis of zucchini fruit response to low temperature revealed several molecular mechanisms involved in chilling tolerance and identified candidate genes that could regulate or promote chilling tolerance, including the transcription factors *MYB76*-like, *ZAT10*-like, DELLA protein GAIP, and AP2/ERF domain-containing protein [18]. Previous research identified 132 *AP2/ERF* genes of winter rapeseed exposed to low-temperature stress with transcriptome sequencing [16]. Gene Ontology (GO) functional analysis and promoter sequence analysis revealed that these genes are involved in many molecular pathways that may enhance cold resistance. Basnet conducted a global microarray gene co-expression analysis by measuring the transcript abundance of developing seeds from two diverse *Brassica rapa* morphotypes, which showed that the seed development processes in *Brassica rapa* is at later developmental stages than in the related species *Brassica napus* [21].

Winter rapeseed is the only oilseed crop in northern China that can safely and stably overwinter, and the seed yield and oil content of winter *Brassica rapa* in this region is greater than that of flax (*Linum usitatissimum* L.) and other oilseed crops [2]. Although the physiology, proteomics, and micRNA of cold tolerance of winter *Brassica rapa* have recently been studied, the complex mechanism of cold tolerance is still unclear [22,23]. The aboveground parts of winter *Brassica rapa* wither during winter, whereas the roots and growing point are capable of overwintering and the growing point likely plays a key role in tolerance to low temperatures [24]. Unlike wheat and other overwintering plants, cold tolerance in winter *Brassica rapa* has rarely been studied. 

In the present study, transcriptome profiles in cold-tolerant versus cold-sensitive winter *Brassica rapa* were investigated using RNA-seq. This study aims to explore novel genes related to the cold tolerance of winter *Brassica rapa* and to provide the molecular genetic basis for breeding winter *Brassica rapa* with cold tolerance.

## 2. Results 

### 2.1. Morphological and Physiological Responses to Cold Stress

**Figure 1:**
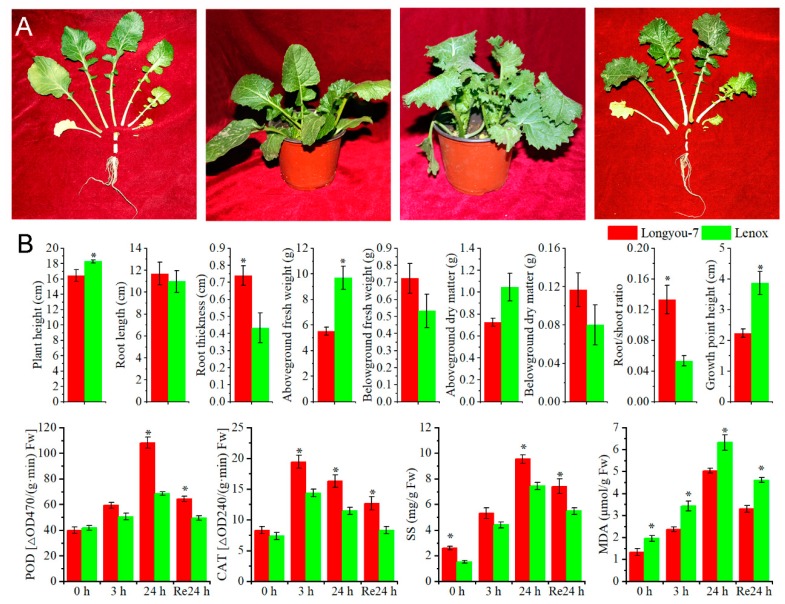
Plant growth, morphological, and physiological indicators. (**A**) Growth characteristics of Longyou-7 and Lenox under cold stress at 4 °C; (**B**) Morphological and physiological indicators of Longyou-7 and Lenox under cold stress at 4 °C. Error bars denote standard error of the mean. Significant differences between varieties at *p* ≤ 0.05 are denoted by an asterisk. Abbreviations: CAT: Catalase, MDA Malondialdehyde, POD Peroxidase, SS Soluble sugar.

Seedling morphological characteristics and physiological responses of Longyou-7 and Lenox were used to study cold stress. Longyou-7 injury was minimal after 24 h of cold stress, while the leaves of Lenox were damaged due to injury (Figure 1A). Root thickness and root‒shoot ratio of Longyou-7 were significantly greater than those of Lenox (Figure 1B). The degree of cold tolerance was assessed based on peroxidase (POD) activity, catalase (CAT) activity, soluble sugar (SS) content, and malondialdehyde (MDA) content, and was significantly increased in the growth point of both two varieties under cold stress (Figure 1B). POD activity, CAT activity, soluble sugar content, and MDA content of the two varieties increased under cold stress at 4 °C, and POD activity, CAT activity, and SS content of the growing point of Longyou-7 were greater than that of Lenox. However, Lenox had greater MDA content than longyou-7. After 24 h of room-temperature recovery, the content of each indicator decreased (Figure 1B).

### 2.2. Transcriptome Analysis and Alignment of Unique Reads with Brassica rapa Reference Genome 

The RNA-seq of Longyou-7 and Lenox was compared to identify key differential genes that respond to cold stress. Twenty-four libraries from eight samples (three biological replicates for each sample) were constructed (Table 1). Each library yielded approximately 50 million raw reads (a total of 1248.17 million raw reads). In view of the impact of the data error rate on the results, Trimmomatic software was used for quality preprocessing of the raw data [25]. After removing low-quality reads, 1206.54 million clean reads were obtained, with an average of 50.27 million clean reads per library, and the Q30 for all libraries was greater than 92%, and the GC content of each treatment was approximately 48%. About 85% of clean reads mapped to the reference genome. Multiple mapped clean reads in each library were excluded from further analysis. A total of 36.97 to 43.59 M uniquely mapped clean reads were used for subsequent analysis. The distribution of unique reads with chromosome ‘+/−’ chain and splice/non-splice in each library were counted (Table S1). The FPKM density profile is a non-standard normal distribution with a regional area size of 1, representing a sum of approximately 1 for probability. The correlation coefficient of gene expression level between samples was close to 1, and the similarity of expression patterns between samples was high, indicating that the experiment was reliable and the sample selection was reasonable (Figure S1).

### 2.3. Identification of Differentially Expressed Genes with Cold Stress

**Figure 2:**
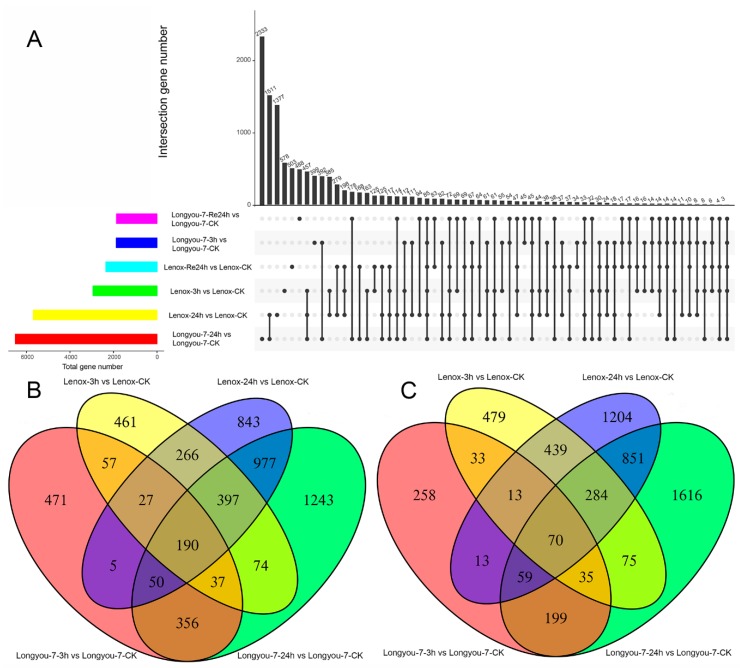
DEGs identified for three treatments in Longyou-7 and Lenox. (**A**) All DEGs for the three treatments. The bar charts indicate DEGs under single treatment (the *x* axis represents the number of genes); column charts indicate DEGs under single or multiple processes (in the *x*-axis, black dots represent a single treatment, black lines connected by dots represent multiple treatments, and the *y*-axis represents the number of genes corresponding to them). (**B**) Upregulated DEGs identified with cold treatment at 4 °C for 3 and 24 h in Longyou-7 and Lenox; (**C**) downregulated DEGs identified with cold treatment at 4 °C for 3 and 24 h in Longyou-7 and Lenox.

**Figure 3:**
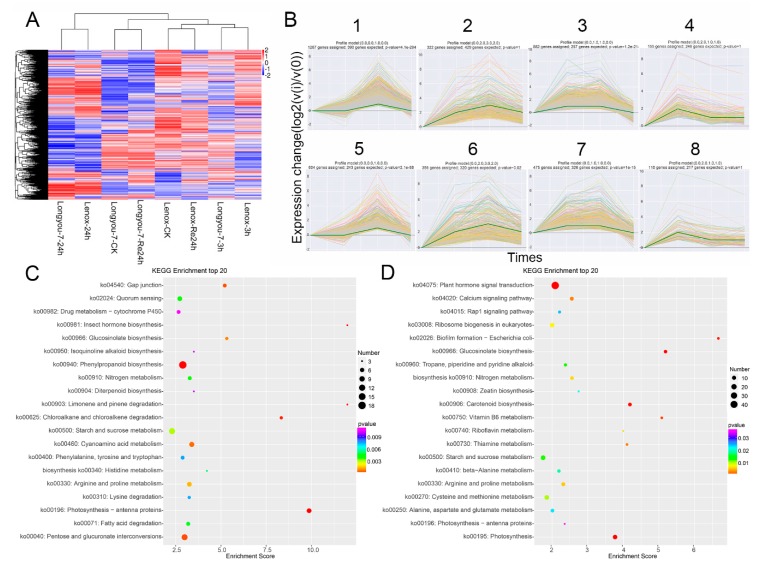
Expression profiles of DEGs for different treatments and annotations of the KEGG pathway. (**A**) Based on the log2-fold-change value of the comparison process and the control, hierarchical clustering of DEGs was performed for different treatments to obtain eight clusters; (**B**) eight representative expression patterns of eight clusters were screened; (**C**) KEGG-enriched DEGs in Longyou-7 at 3 and 24 h of cold stress; (**D**) KEGG-enriched DEGs in Lenox at 3 and 24 h of cold stress.

To comprehensively investigate differences in gene expression between cold-sensitive and cold-tolerant rapeseed in response to cold stress, FPKM was used to calculate gene expression, and based on the criteria of *p* < 0.05 for adjusted *p*-value and greater than 1 for log2 base of fold-change, a total of 10,251 and 10,972 DEGs were identified in Longyou-7 and Lenox, respectively (Figure 2A, Table S2). The Venn map reflects the up- and downregulation of DEGs in two winter rapeseed varieties under cold stress (Figure 2B,C). After 3 h of cold stress, a total of 462 (311 up- and 151 downregulated) DEGs were identified in rapeseed, respectively (Figure 2B,C, Table S3). After 24 h of cold stress, a total of 2878 (1614 up- and 1264 downregulated) DEGs were identified in winter rapeseed (Figure 2B,C, Table S3), 127 (86 up- and 41 downregulated) and 179 (109 up- and 70 downregulated) are related to response to cold, response to freezing, response to temperature stimulus, cold acclimation, cellular response to freezing, and cellular response to cold genes were identified in Longyou-7 and Lenox, respectively (Table 2, Table S4). Out of the 127 DEGs, 46 cold-responsive genes (25 up- and 21 downregulated) were exclusively identified in Longyou-7, whereas 98 cold-responsive genes (48 up- and 50 downregulated) were uniquely observed in Lenox. The remaining 81 genes (61 up- and 20 downregulated) were commonly regulated by cold stress in both Longyou-7 and Lenox. However, after 24 h of recovery at room temperature, there was still some differential expression of cold-resistant genes. The small number of cold-reacting genes identified in the cold-tolerant Longyou-7 indicates that itself has strong cold tolerance and is not sensitive to cold stress. To compare the transcriptomes in Longyou-7 and Lenox under cold treatment, a heat map was generated to present the transcript abundance for all DEGs under treatments. After cold stress, a series of changes occurred in the expression of genes for Longyou-7 and Lenox (Figure 3A). In addition, these DEGs were induced in Lenox much less than Longyou-7, such as *Adenosylhomocysteinase 2* (Bra028345) (Figure 3A). This may be one of the main reasons why Longyou-7 is more resistant to cold than Lenox.

According to their expression profiles, the differentially expressed genes of the two varieties were clustered into 27 groups (Figures S2 and S3). For the upregulated expression profiles of the four groups of three treatments in Longyou-7 (Figure 3B), groups 1 and 2 contained the most DEGs induced by 24 h of cold stress (1267 and 322, respectively), and groups 3 and 4 contained the most DEGs induced by 3 h of cold stress (822 and 155, respectively). For the upregulated expression profiles of the four groups of three treatments in Lenox, groups 5 and 6 contained the most DEGs induced by 24 h of cold stress (604 and 356, respectively), and groups 7 and 8 contained the most DEGs induced by 3 h of cold stress (475 and 110, respectively).

**Figure 4:**
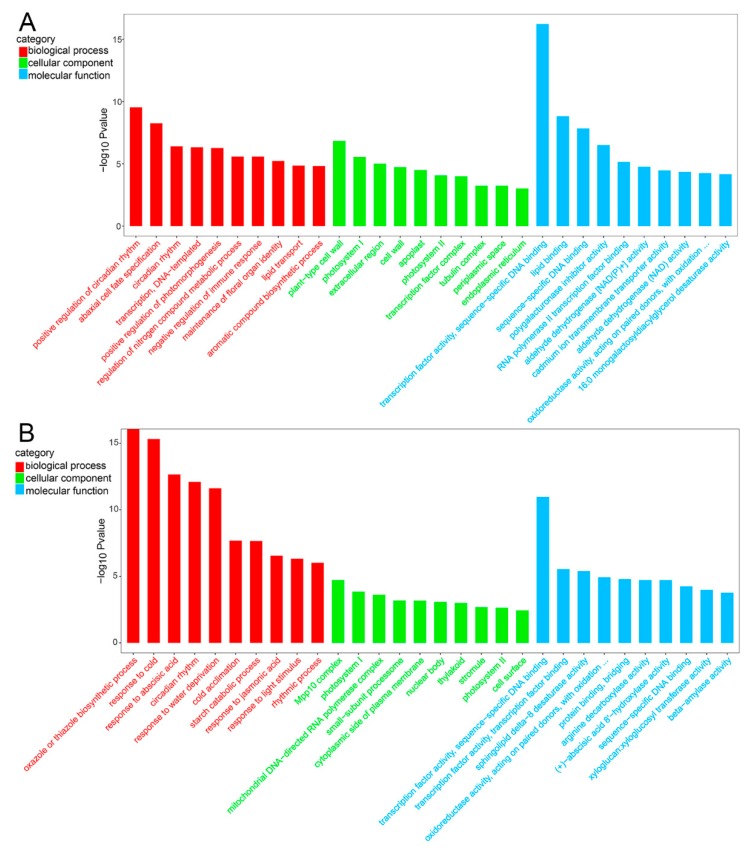
Visualization of GO enrichment terms related to cold response. Functional classification of GO terms obtained using WEGO software and summarized using REVIGO. Terminology associated with DEGs with 3 and 24 h of cold treatment in Longyou-7 (**A**) and Lenox (**B**).

Analysis of DEGs in two varieties attempted to study the mechanisms that play a role in the cold stress response. Functional classification of DEGs was achieved using GO analysis. These DEGs are divided into three categories of GO: biological process, molecular function, and cellular component (Figures S4 and S5). The top 30 of GO enrichment analysis (screening GO entries corresponding to the number of differential genes greater than 2 in the three categories, each of the 10 sorted by -log10 *p*-value for each entry) was used to screen DEGs under cold stress in Longyou-7 and Lenox, respectively. Higher GO terms are “transcription factor activity,” “lipid binding,” “plant-type cell wall,” “positive regulation of circadian rhythm,” and “abaxial cell fate specification” in Longyou-7 (Figure 4A, Table S5). In Lenox, higher GO terms are “oxazole or thiazole biosynthetic process,” “response to cold,” “response to abscisic acid,” and “circadian rhythm” (Figure 4B, Table S5).

The KEGG (Kyoto Encyclopedia of Genes and Genomes) pathway analysis provides classification of the complex biological functions of the research genes [26]. Top20 of KEGG enrichment analysis (screening for pathway entries with a number of differential genes greater than 2, sorted by -log10 *p*-value for each entry) showed that “ko00940: phenylpropanoid biosynthesis,” “ko00040: pentose and glucuronate interconversions,” and “ko00500: starch and sucrose metabolism” were significantly enriched by DEGs under cold stress in Longyou-7 (Figure 3C, Table S6), and that “ko04075: plant hormone signal transduction,” “ko00500: starch and sucrose metabolism,” and “ko00195: photosynthesis” were significantly enriched by DEGs under cold stress in Lenox (Figure 3D, Table S6). 

### 2.4. Analysis of Transcription Factors and SNP/INDEL

**Figure 5:**
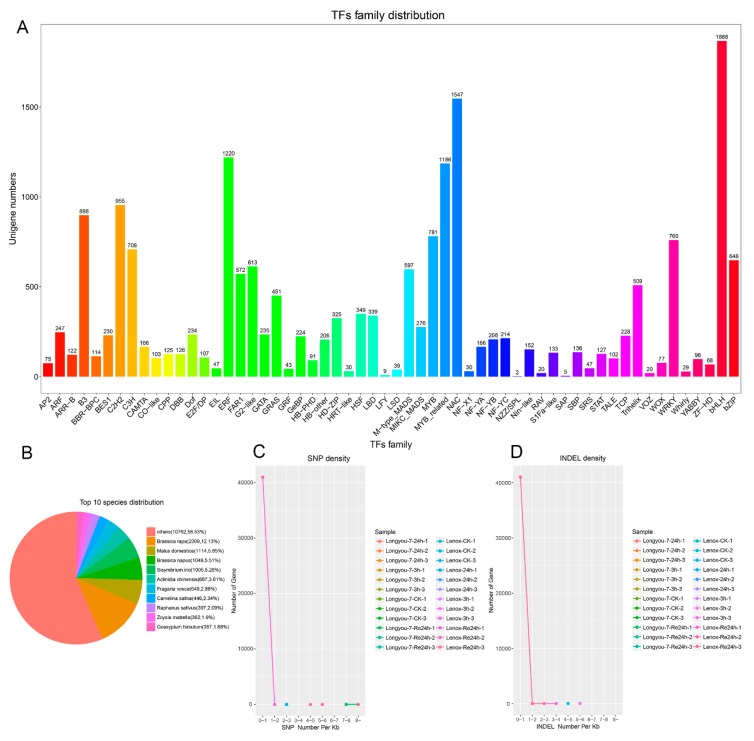
Summary of statistical analysis of transcription factors (TFs), SSRs, and SNPs with cold treatment. (**A**) 58 differentially expressed TFs; (**B**) identified TFs clustered into top 10 species distributions; (**C**) SNP density distribution map; the horizontal axis is the number of SNPs per 1000 bp sequence on the gene and the vertical axis is the number of genes; (**D**) INDEL density map; the horizontal axis is the number of INDELs per 1000 bp sequence on the gene and the vertical axis is the number of genes.

Transcription factor (TF) is the key to regulation of gene expression under abiotic and biotic stress in plants. The RNA-seq results show that many TFs were regulated under cold stress of Longyou-7 and Lenox. There were a total of more than 58 families of TF differentially expressed in winter *Brassica rapa* (Figure 5A). This study identified and blasted 19,037 TFs in the data bases and clustered them into the top 10 species distribution. *Brassica rapa* matched 2309 transcription factors, accounting for 12.13% (Figure 5B). Families sensitive to cold treatment included bHLH, NAC, ERF, and MYB_related; these transcription factor families have more gene expression changes that play an important role in regulating the genes involved in cold response (Table S7).

For further application of winter *Brassica rapa*, SNPs and INDELs were discovered using the assembled transcriptomes. A total of 833,567 SNPs was detected in transcriptomes, among which 487,704 were transitions, and 345,863 were transversions. The number of SNPs in Longyou-7 with strong cold resistance was less than that of Lenox with weak cold resistance. Most SNPs were distributed in the exon region and a few were distributed in the upstream region (Figure 5C, Table S8). A total of 48,149 and 51,090 INDELs were detected in Longyou-7 and Lenox, respectively. Most of the INDELs were distributed in the upstream region and a few were distributed in the exon and downstream regions (Figure 5D, Table S8). The SNPs and INDELs identified in this study provide valuable resources for future genetic linkage mapping studies and specific trait analysis in winter *Brassica rapa*. 

### 2.5. Validation of Differential Genes by Quantitative RT-PCR Analysis

**Figure 6:**
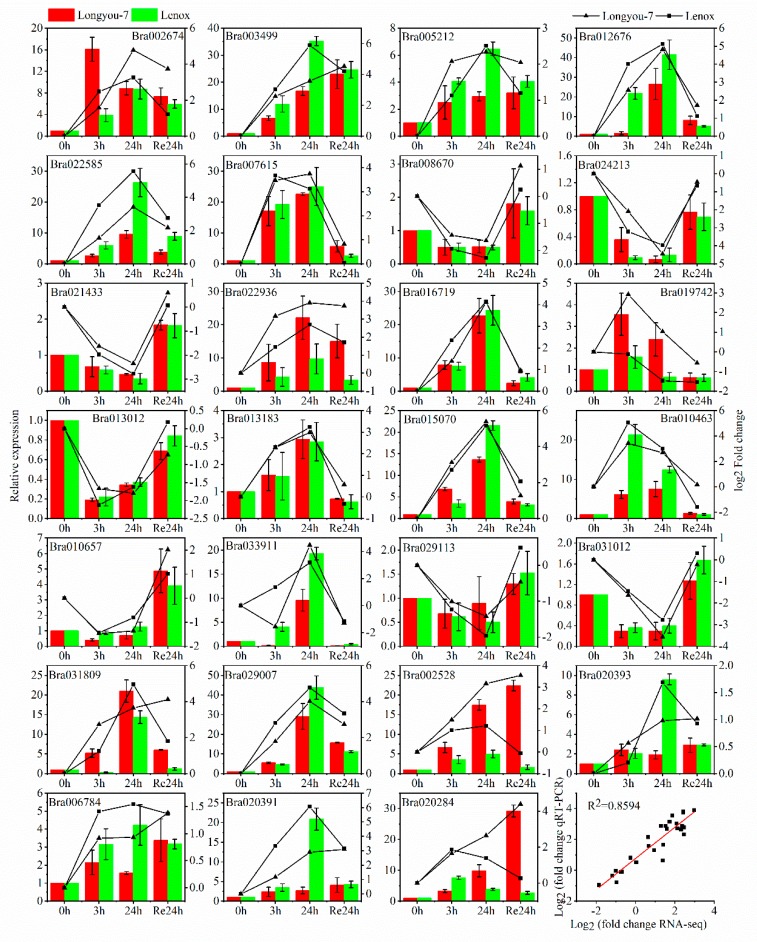
qRT-PCR analysis of 27 cold-induced unigenes during various stages of cold stress. The expression pattern of the selected gene was analyzed by treating at 4 °C for 0, 3, and 24 h and recovery at 22 °C for 24 h. Bars with standard errors represent relative expression levels determined by qPCR from three independent biological replicates using the 2^−ΔΔCT^ method (left *y*-axis). Broken lines indicate transcript abundance change (log2 fold) according to the RPKM value of RNA-Seq (right *y*-axis). Correlation between qRT-PCR and RNA-seq for select DEGs is also shown.

Some genes associated with cold response and genes with high differences in transcriptome expression were selected for qRT-PCR. The quantitative RT-PCR results from Longyou-7 and Lenox at 4 °C for 0, 3, and 24 h, and Re24 h are shown in Figure 6, and the expression profiles of all 27 detected genes show the same trend and consistent results between RT-PCR and RNA-seq (*r*^2^ = 0.859). For 3 and 24 h of cold stress, seven DEGs displayed the same downregulation in both winter rapeseed varieties: Bra008670 (*Basic leucine zipper 43*), Bra024213 (*E3 ubiquitin‒protein ligase RMA2*), Bra021433 (*LOB domain-containing protein 41*), Bra013012 (*Probable acyl-activating enzyme 17, peroxisomal*), Bra010657 (*Ethylene-responsive transcription factor ERF060*), Bra029113 (*Transcription factor MYB82*), and Bra031012 (*12-oxophytodienoate reductase 2, N-terminally processed*). However, two DEGs showed opposite changes in Longyou-7 and Lenox: Bra019742 (*Lipid transfer protein EARLI 1*) and Bra031809 (*Dehydrin Rab18*). Strikingly, there was a remarkable difference of *temperature-induced lipocalin-1* and *zinc finger protein ZAT12* expression profile between the two winter rapeseed varieties in cold stress. *Temperature-induced lipocalin-1* (Bra002674) of Longyou-7 was significantly upregulated to ~5-fold that of Lenox in early cold stress (3 h), and was greater than Lenox until 24 h (Figure 6); however, the transcripts of the other three *temperature-induced lipocalin-1* (Bra020393, Bra006784, and Bra020391) in Longyou-7 were downregulated more than those of Lenox. The *zinc finger protein ZAT12* (Bra002528 and Bra020284) was upregulated (3.5- and 3-fold, respectively) compared to Lenox at 24 h of cold stress (Figure 6), and after 24 h of recovery at room temperature Longyou-7 also had significantly upregulated expression that was 14 and 11 times that of Lenox, respectively. Moreover, Bra020284 in Lenox began to downregulate after 3 h of cold stress, which was opposite to that of Longyou-7. We conclude that the qRT-PCR results validate the transcriptomic profiling data obtained from our RNA-seq analysis.

## 3. Discussion

Chilling and freezing damage are common environmental factors faced by many crops in northwestern China, severely restricting the type of crops produced [27]. Sun et al. studied the feasibility of expanding winter rapeseed (*Brassica rapa*) to the northwest and cold and arid regions of North China, and made breakthroughs in cold-resistant breeding. Winter rapeseed varieties of strong cold resistance can now be safely produced in Xinjiang, Tibet, Heilongjiang, and the most northern regions of China. Winter rapeseed has become a new overwintering crop in these areas, with a rapid expansion of planting area and significant economic and ecological benefits [2,28]. It is important to identify the cold resistance mechanism of winter rapeseed (*Brassica rapa*). Stress breaks the redox and energy balance in plants [29]. Exposure of plants to low temperatures causes the accumulation of reactive oxygen species (ROS), such as hydrogen peroxide (H_2_O_2_), singlet oxygen (O_2_^1^), superoxide anions (O_2_^−^), and hydroxyl radicals (·OH), causing oxidative damage in plants [30]. Under stress, plants evolved to form macromolecules that act as osmotic regulators and play an important role in maintaining cell‒liquid osmotic balance [30,31]. Xia et al. showed that ROS is a signaling molecule involved in the response of cucumbers to low-temperature and paraquat stress [32]. MDA is a product of lipid peroxidation and the extent of membrane damage can be reflected by MDA levels in stressed tissues [33]. POD can maintain balance by removing excess ROS, which helps plants to endure stress conditions [34]. The content of MDA and O_2_^−^ in the growth point of Lenox was greater than that of Longyou-7 after cold stress, while the protective enzyme activity and soluble regulator content of Longyou-7 were greater than that of Lenox. Aboveground tissues of winter rapeseed were damaged by low temperatures. Longyou-7 has strong protective enzyme activity and high content of soluble regulators, which can remove ROS.

Many metabolomics [12,35], functional genomics [36,37], high-throughput sequencing [23], and proteomics [38] studies have identified the molecular mechanisms of plant cold tolerance. However, the current understanding of the complex mechanisms of winter rapeseed resistance to cold is still limited. In this study, transcriptome sequencing was used to elucidate the cold tolerance mechanism in response to cold stress in winter rapeseed. We calculated that the number of unique alignments of each sample sequencing sequence on the reference sequence was more than 82%, and that nearly 18% of the sequencing sequences failed to match the reference gene. The FPKM method was used to compare gene expression differences between different samples [39] and some of the differential genes were not described. Further gene function and expression studies are required to identify key genes for cold resistance of winter rape (Tables S1 and S9). We analyzed the amount of DEGs by comparing the tolerant Longyou-7 and the sensitive Lenox at 3 and 24 h of cold treatment, and at 24 h of recovery following cold treatment, and in non-treated control conditions. The number of DEGs was greater in Longyou-7 than Lenox, and at 24 h of cold stress there were 569 more DEGs in Longyou-7 than Lenox. However, for the cold treatment of 3 h, Lenox upregulated 316 more DEGs than Longyou-7 and downregulated 748 more DEGs than Longyou-7 (Table S2). These results may indicate that the common DEGs in the cold-sensitive winter rape in the early stage of cold stress at 4 °C are expressed earlier, while in tolerant winter rapeseed, some DEGs are upregulated in the late stage of cold stress. Interestingly, some genes were still upregulated after 24 h of recovery at room temperature and showed different performance in both varieties (Table S3) [40]. The two varieties also had different expression patterns of specific DEGs and different systems of cold tolerance. Previous research has shown that rapid activation and selective induction of cold-tolerant pathways in canna and other cold-specific genes may be among the main reasons for the greater cold resistance of plantains compared to bananas [1].

A transcriptome study related to cold stress was previously performed on other crops, and the GO term ‘response to stimulus’ was emphasized by GO analysis, from which some cold stress genes were identified [14,41]. The GO analysis in this study showed that six terms closely related to cold stress were annotated, including cellular response to cold (GO:0070417), cellular response to freezing (GO:0071497), cold acclimation (GO:0009631), response to cold (GO:0009409), response to freezing (GO:0050826), and response to temperature stimulus (GO:0009266) (Table S5). However, at 24 h of cold stress, many genes encoding a cold stress response showed increased transcript abundance, and the transcripts associated with cold stress response to Lenox at 3 h were greater than Longyou-7. With 24 h of cold stress, some ‘response to cold’ terms were significantly enriched in the upregulated genes of the two varieties. At the same time, these genes were expressed greater in Longyou-7 than Lenox, including *temperature-induced lipocalin-1* (Bra002674), *zinc finger protein ZAT12* (Bra020284, Bra002528), *dehydrm ERD10* (Bra025819), and *adenosylhomocysteinase 2* (Bra028345) (Figure 6). Studies have shown that *temperature-induced lipocalin* plays an important role in combating cold stress and abiotic stress in *Arabidopsis* and *Medicago falcata* [42,43]. Research has also shown that function of the highly conserved ERD4 protein may be associated with its RNA-binding ability during stress response. The functional annotation of the ERD4 family of proteins that can be useful in designing experiments to unravel crucial aspects of stress tolerance mechanism [44]. In *Arabidopsis*, constitutive expression of *Zat12* leads to enhanced expression of oxidative stress and light stress transcripts, and *Zat12* plays an important role in ROS and abiotic stress signaling [45]. The results indicate that the response to cold-related genes in cold regulation mechanism may contribute to the cold tolerance of winter rapeseed.

**Figure 7:**
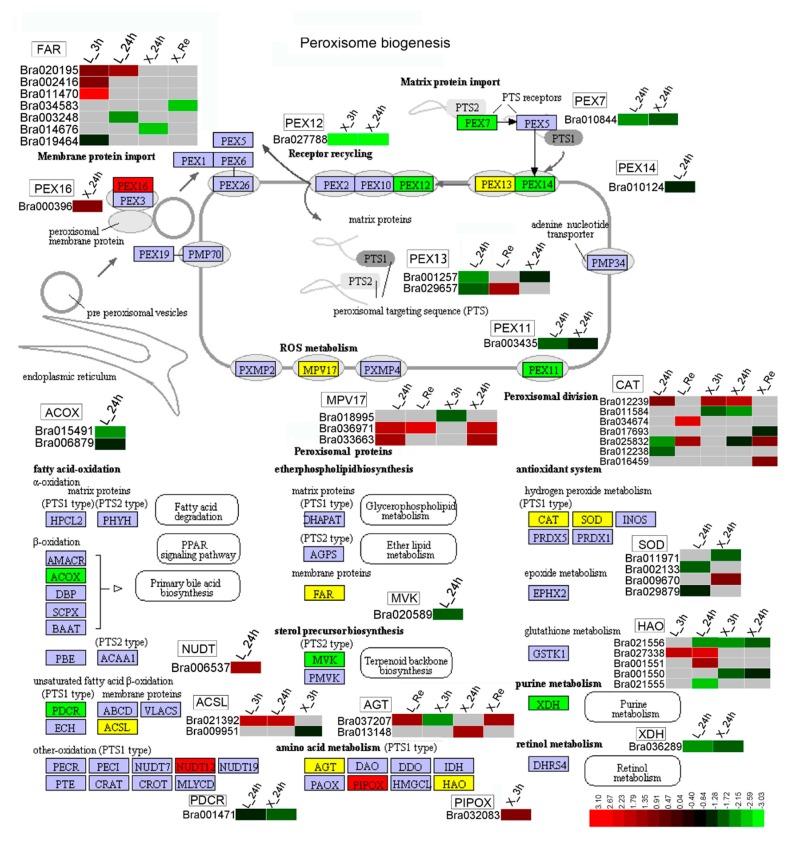
Cold-induced genes associated with the peroxisome pathway. Gene expression values were mapped to the reference pathway using KEGG. Red on the access map shows upregulated genes, green indicates downregulated genes, and yellow indicates genes that are both upregulated and downregulated. Gene annotations by KEGG in RNA-seq are represented in a heat map, where red indicates upregulated genes, green indicates downregulated genes, gray indicates no KEGG annotation, and DEGs for each pathway are marked next to the pathway map.

KEGG enrichment analysis was performed on all DEGs in Longyou-7 and Lenox growth points. The peroxisome (brp04146) pathway was significantly enriched at 24 h of cold stress (Table S6). Cold exposure of antioxidant enzymes and antioxidant-associated genes often induces oxidative stress in plants. To counteract excess ROS formed in the peroxisome or cytosol, the mitochondria (in the UQ/UQH2 cycle), activates the plant’s internal antioxidant system consisting of scavenging enzymes and antioxidants [46,47]. In this study, the *PXMP2/4 family protein 4* (*MPV17*) directly cleared excess ROS and the expression of Longyou-7 was significantly greater than that of Lenox after cold stress, and *Peroxisomal (S)-2-hydroxy-acid oxidase* in Longyou-7 (Figure 7). The expression of *GLO1* (*HAO1*) and *long chain acyl-CoA synthetase 1* (*ACSL1*) increased by 1.84, 2.32, 2.19, and 2.03 (log2 fold-change) and three transcripts of *fatty acyl-CoA reductase 1* (*FAR1*) were upregulated in Longyou-7 after 3 and 24 h, respectively. However, KEGG did not annotate these transcripts in Lenox. These results reflect that there may be different metabolic patterns in the peroxisome pathway of Longyou-7 and Lenox. The tolerance of Longyou-7 to chilling injury is related to the enhancement of antioxidant enzymes under cold stress [48]. In the present study, 31 and 26 genes of the peroxisome pathway were enriched in Longyou-7 and Lenox, respectively. A total of 13 of these two genes were co-expressed in both genotypes, most of which were expressed to a greater degree in Longyou-7 than Lenox at three time points, and the results indicated that the metabolic activity of peroxisome in Lenox was enhanced. In addition, we found that photosynthesis-antenna protein (brp00196), cyanoamino acid metabolism (brp00460), basal transcription factors (brp03022), and plant hormone signal transduction (brp04075) pathways in Lenox were significantly enriched by KEGG. These transcripts play an important role in plant resistance to cold stress [1,12,14].

It has been reported that APETALA2/ethylene response factor (AP2/ERF) plays an important regulatory role in the signal transduction of rapeseed to low temperature [16]. The 14-3-3 family of ubiquitous proteins exhibited significant up- or downregulated expression under cold and heat stress, revealing their potential role in the regulation of abiotic stress responses [49]. The WRKY protein contains a large number of transcription factors and plays an important role in many aspects of physiological processes and environmental adaptation [50]. In the present study, UDP-glycosyltransferase 84A2 (NF-YB) was identified in both Longyou-7 and Lenox after 24 h of cold treatment and that expression was significantly greater in Longyou-7, while it was only found in Lenox after 3 h of cold treatment. With cold treatment, transcription factor bHLH150 (bHLH) was upregulated in both varieties, and the expression in Lenox was greater than that in Longyou-7 at 3 h and 24 h (Tables S3 and S7). These results indicate that transcription factors in sensitive winter rapeseed are more susceptible to activation under cold stress, while tolerant winter rapeseed was slower to respond to cold stress. More transcription factors need to be identified, and cold-resistant winter rapeseed may have unique genes responsive to cold stress.

## 4. Methods

### 4.1. Plant Material, Cold Treatments, and Morphological and Physiological Analyses

*Brassica rapa* cultivars Longyou-7 and Lenox from Gansu Agricultural University were used in this study. For the RNA-seq experiments, seeds were germinated and cultivated in plastic pots under typical conditions until the plants grew to the six-leaf stage at Gansu Agricultural University in Lanzhou, China. Control plants were then grown in a growth chamber under normal conditions (22 °C with 16/8 h light/dark cycle) and morphological indicators (plant height, root length, steam diameter, aboveground fresh weight, belowground fresh weight, aboveground dry matter, belowground dry matter, root/shoot ratio, growth point height) were recorded. For cold treatments, plants were transferred to a 4 °C growth chamber (Yuejin, Shanghai, China). The growth points of plants subjected to 22 °C (as control, CK) and 4 °C for 3 h, 24 h, and recovery at 22 °C for 24 h (as treatments, TR) were collected and frozen in liquid nitrogen and stored at −80 °C, then used for RNA extraction. Each sample were pooled from three plants and three biological replicates were included. POD (peroxidase) activity, CAT (catalase) activity, SS (soluble sugar) content, and MDA (malondialdehyde) content were analyzed as indicators of physiological response [51,52,53]. 

### 4.2. RNA Isolation, Library Construction, and Illumina Sequencing

Total RNA was extracted using TRIZOL (Invitrogen, Carlsbad, CA, USA) according to the manufacturer’s protocol for each biological replicate. Total RNA was extracted from each sample and DNA was digested with DNase; eukaryotic mRNA was enriched with magnetic beads with Oligo (dT), and mRNA was broken into short fragments by an interrupting reagent. The mRNA was used as a template and a single-strand cDNA was synthesized using a six-base random primer. Then, a two-stranded cDNA was synthesized to a double-stranded cDNA that was purified using a kit (Invitrogen, Carlsbad, USA); the purified double-stranded cDNA was subjected to end repair and a tail was added. The sequencing adaptor was ligated, then the fragment size was selected and PCR amplification was performed. The constructed library was qualified using Agilent 2100 Bioanalyzer and sequenced using an Illumina HiSeq X Ten sequencer (Shanghai, China) to generate 150 bp double-ended data (NCBI accession number: SRP179662).

### 4.3. Assembly, Data Analysis, and Functional Annotation

The *Brassica rapa* genome with high homology was chosen as the reference genome, the Ensembl Genomes database (Hinxton, Cambridgeshire, UK) was used for blast, and Trimmomatic software (Aachen, Germany) was used for quality preprocessing of raw data [25]. Hisat2 (Baltimore, MD, USA) was used to sequence clean reads with the specified reference genome, obtain positional information on the reference genome or gene, and sequence characteristic information specific to the sequenced sample [54]. Htseq software (Heidelberg, Germany) was used to obtain the number of reads on the gene in each sample [55] and cufflinks software was used to calculate gene expression FPKM values [56]. Pearson’s correlation coefficient was used to evaluate the linear association between gene expression levels of sequencing samples. Principal component analysis of gene expression levels was performed to calculate the distance between samples using clustering method. The number of counts of each sample gene was normalized using DESeq software with the base mean used to estimate expression level, the difference was calculated, and statistical significance was assessed using the negative binomial distribution test [56]. RNA-seq means from the same gene in two samples were considered statistically significant when there was a fold change greater than 2 and when the adjusted *p*-value was less than 0.05. 

GO enrichment analysis of differentially expressed genes was conducted by pathway analysis using the KEGG database [57]. Based on assembly results of reads by StringTie software (Baltimore, MD, USA) [58], the existence of variable shear events in the sample was detected using ASprofile software (Baltimore, USA) [59]. Samtools software (Beijing, China) was used for chromosome coordinate sorting and de-weighting [60], bedtools software (San Francisco, CA, USA) was used to predict SNP and INDEL sites in the sample [61], and snpEff software (San Francisco, CA, USA) was used to comment on the function [62].

### 4.4. Quantitative Real-Time PCR Validation

Twenty-seven transcripts were selected for quantitative real-time PCR (qRT-PCR) analysis to verify the reliability of transcriptome sequencing. One microgram of total RNA was used for first-strand cDNA synthesis according to the protocol supplied with PrimeScript™ RT Master Mix (TaKaRa Biotechnology Co., Ltd., Dalian, China) and qRT-PCR amplification reactions were performed using a LightCycler®96 Real-Time PCR System (Roche, Basel, Switzerland). Primers used for qRT-PCR assay were designed by Primer-BLAST (http://www.ncbi.nlm.nih.gov/tools/primer-blast/) (Bethesda, MD, USA) based on the sequences of the selected unigenes (Table S10). Mean values and standard errors were calculated from three independent experiments with three biological replicates, and relative expression was calculated using the 2^−ΔΔCt^ method with *Actin* as the reference gene [23].

## 5. Conclusions

In this study, two winter cultivars (Longyou-7 and Lenox) with different phenotypes for cold stress were used for transcriptome sequencing to analyze their molecular mechanisms for cold response. A total of 10,953 upregulated and 10,270 downregulated core DEGs were identified in the growth point of winter rapeseed (*Brassica rapa*) at 4 °C for 3 and 24 h, and at room temperature for 24 h. A hypothetical model and heat map of key genes were established to show the difference between Longyou-7 and Lenox and identify key genes for winter rape response to cold stress. The results show that the tolerant Longyou-7 was slower to respond to cold stress than the sensitive Lenox. Six terms closely related to cold stress were annotated, including cellular response to cold (GO:0070417), cellular response to freezing (GO:0071497), cold acclimation (GO:0009631), response to cold (GO:0009409), response to freezing (GO:0050826), and response to temperature stimulus (GO:0009266), these genes are thought to play an important role under cold stress. Longyou-7 had more DEGs at 24 h, mainly involved in signal transduction and ribosome biogenesis. In addition, two transcriptome libraries related to cold and recovery were developed for the first time in winter rapeseed. These findings have value for the analysis of cold resistance in *Brassica rapa* and other types of winter rapeseed (*Brassica napus* and *Brassica juncea*).

## Figures and Tables

**Table 1:** Summary of quality preprocessing of RNA sequencing data.

Sample ^a^	Raw reads (M)	Raw Bases (G)	Clean Reads (M)	Valid Bases (%)	Q30 (%) ^b^	GC (%)
L7-24 h	53.26	7.99	51.65	92.65	95.17	48.44
L7-3 h	51.1	7.67	49.57	91.96	94.22	48.76
L7-CK	52.94	7.94	50.72	90.81	93.01	48.15
L7-Re24	52.33	7.85	50.2	89.7	94.3	48.35
Lenox-24 h	52.74	7.91	51.16	92.65	94.95	48.36
Lenox-3 h	51.25	7.69	49.53	91.45	93.01	48.92
Lenox-CK	49.26	7.39	47.75	91.14	93.96	48.25
Lenox-Re24 h	53.18	7.98	51.61	91.61	95.22	48.17

a: the growth points of Longyou-7 and Lenox subjected to 22 °C (as control, CK) and 4 °C for 3 h, 24 h, and recovery at 22 °C for 24 h (as treatments, TR), three replicates per sample. b: the percentage of bases with a Phred value greater than 30 in the raw bases as a percentage of the total base.

**Table 2:** Differentially expressed genes (DEGs) of cold stress in Longyou-7 and Lenox during cold treatment for 3 and 24 h.

		Longyou-7	Unique in Longyou-7	Lenox	Unique in Lenox	Common in Both
DEGs at 3 h	Total	54	35	103	83	20
	Upregulated	49	31	72	54	18
	Downregulated	6	4	31	29	2
DEGs at 24 h	Total	127	46	179	98	81
	Upregulated	86	25	109	48	61
	Downregulated	41	21	70	50	20
DEGs at RE 24 h	Total	85	50	111	76	35
	Upregulated	69	45	48	24	24
	Downregulated	16	5	63	52	11

## Supplementary Materials

Supplementary materials can be found at https://www.mdpi.com/1422-0067/20/5/1071/s1, Table S1: Statistical results of comparison with reference genomes, Figure S1: Sequencing quality detection. A: Density distribution curve. The curves of different colors in the figure represent different samples, the abscissa of the points on the curve represents the logarithm of the corresponding sample fpkm, and the ordinate of the points represents the probability density. B: PCA map. C: FPKM expression amount distribution map. The different colors in the figure represent different ranges of fpkm values, the abscissa is the sample and the ordinate is the number of genes. D: Sample-to-sample cluster analysis results. The abscissa indicates the sample name, the ordinate indicates the corresponding sample name, and the color indicates the correlation coefficient, Table S2: Statistics on the number of differential gene expression, Table S3: All differentially expressed genes (pval-0.05, FC-2), Table S4: Gene Ontology (GO) analysis of differentially expressed cold-related genes of longyou-7 and Lenox, Figure S2: All DEGs expression profiles in Longyou-7 were at four treatments, Figure S3: All DEGs expression profiles in Lenox were at four treatments, Figure S4: GO Level 2 map of all expressed genes and DEGs at 3 h cold stress, Figure S5: GO Level 2 map of all expressed genes and DEGs at 24 h cold stress, Table S5: Go enriched data for all DEGs, Table S6: KEGG enriched data for all DEGs, Table S7: Expression of transcription factors under cold stress, Table S8: Results of SNP and INDEL distribution, Table S9: Summary of gene expression levels of each sample, Table S10: Primer of qRT-PCR.

## Abbreviations

RNA sequencing	RNA-seq
DEGs	Differentially expressed genes
ROS	Reactive oxygen species
CAT	Catalase
MDA	Malondialdehyde
POD	Peroxidase
SS	Soluble sugar
PCA	Principal component analysis
CK	Control
GO	Gene ontology
KEGG	Kyoto Encyclopedia of Genes and Genomes
qRT-PCR	Quantitative real-time polymerase chain reaction
TF	Transcription factor
