# Supplementary figures and images for: Transcriptome Analysis Reveals Key Cold-Stress-Responsive Genes in Winter Rapeseed (Brassica rapa L.)
Source: Int J Mol Sci. 2019 Mar 1;20(5):1071. doi: 10.3390/ijms20051071 (PMC6429191; doi:10.3390/ijms20051071)

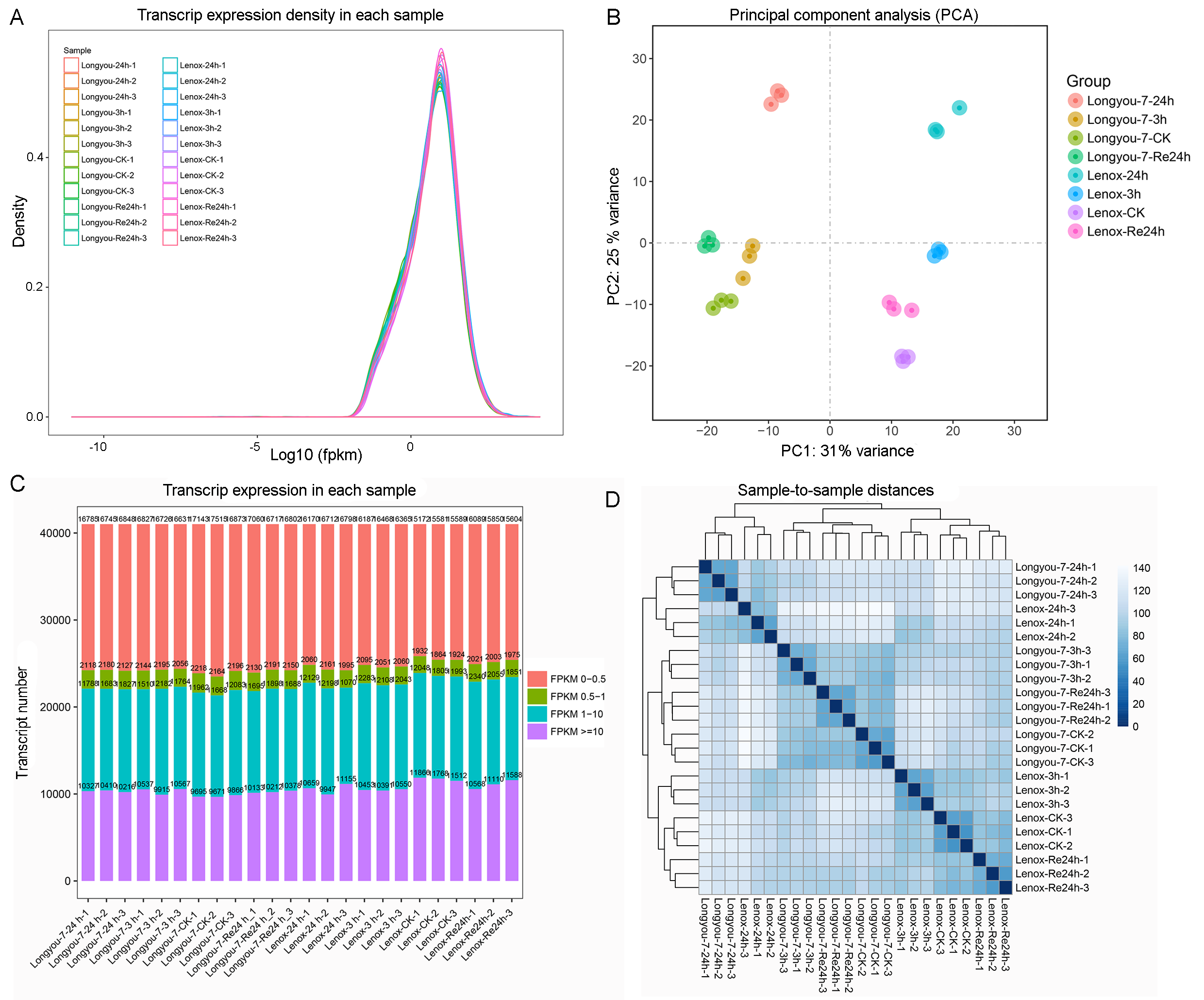

Supplement: Supplementary file 1 [file ijms-20-01071-s001.zip › Figure S1 Sequencing quality detection.tif]

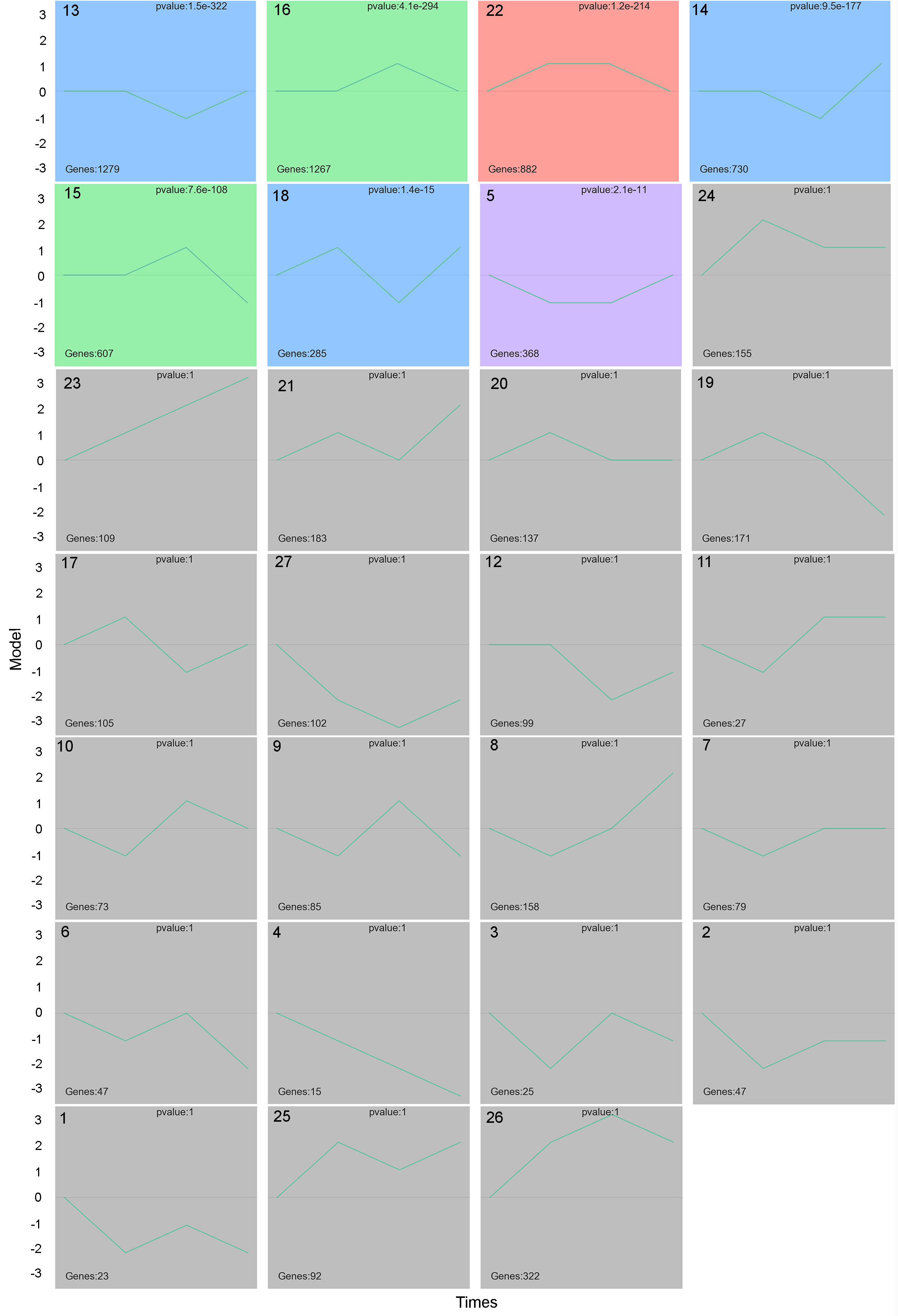

Supplement: Supplementary file 1 [file ijms-20-01071-s001.zip › Figure S2 All DEGs expression profiles in Longyou-7 were at four treatments.tif]

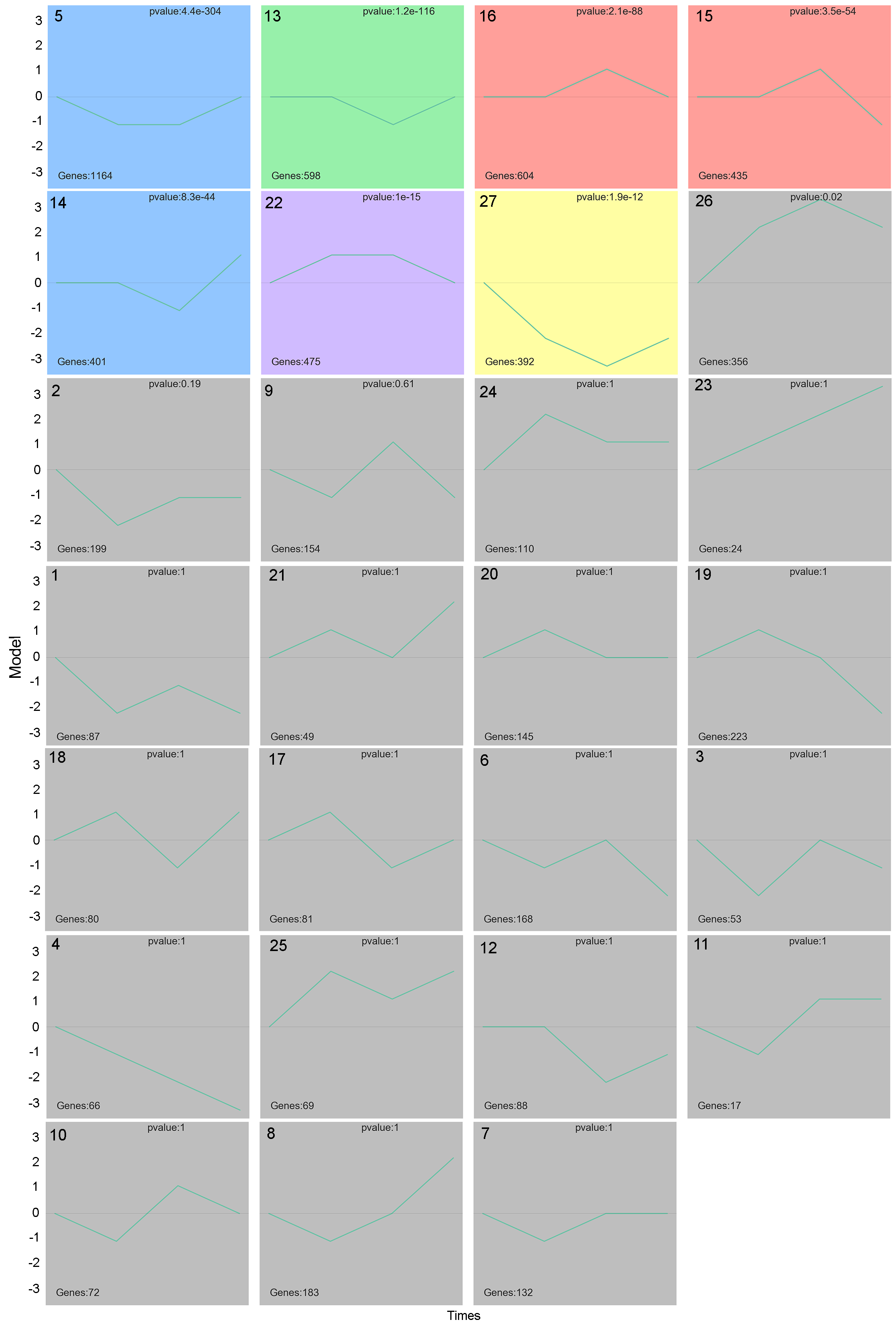

Supplement: Supplementary file 1 [file ijms-20-01071-s001.zip › Figure S3 All DEGs expression profiles in lenox were at four treatments.tif]

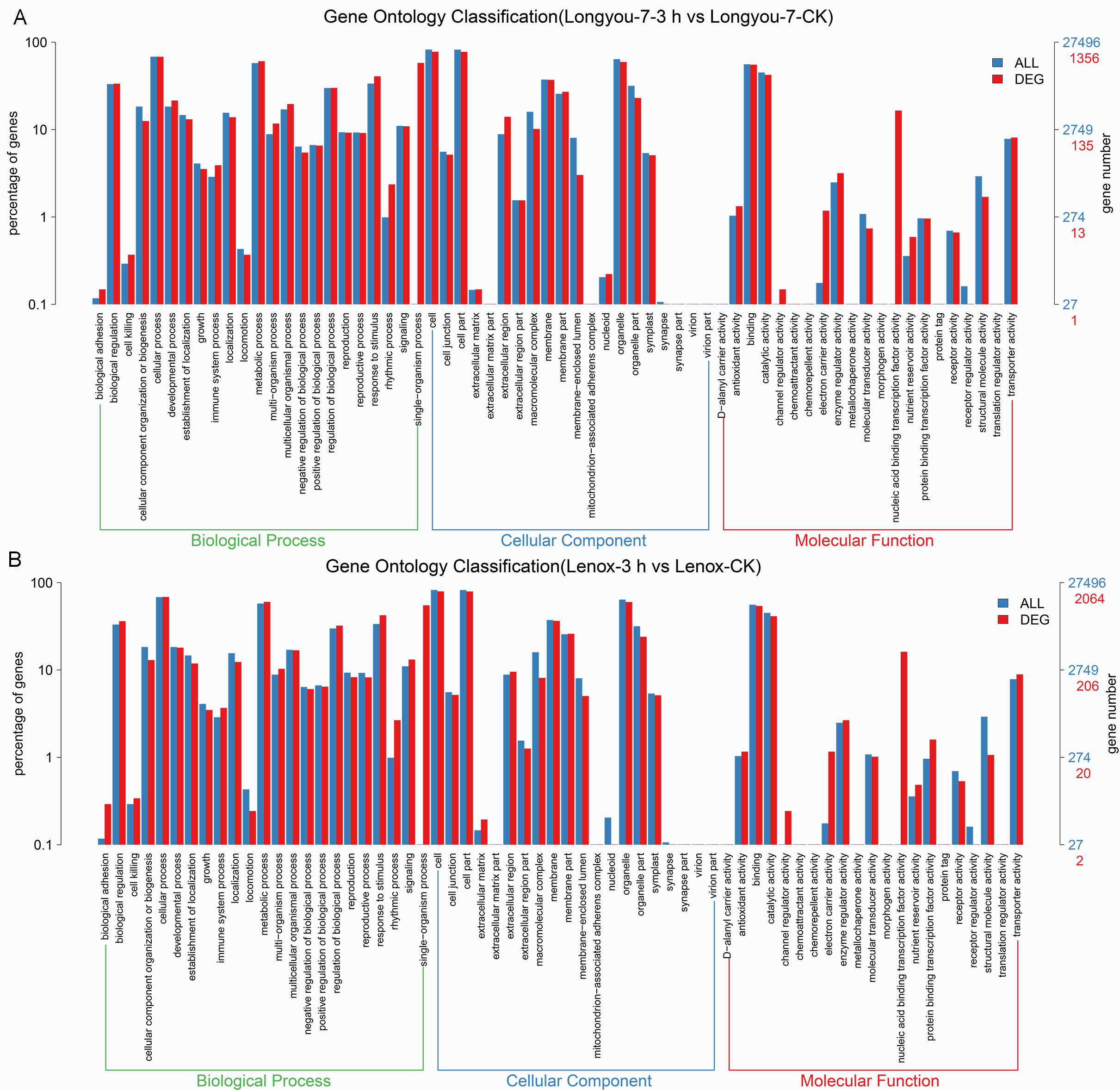

Supplement: Supplementary file 1 [file ijms-20-01071-s001.zip › Figure S4 GO Level 2 map of all expressed genes and DEGs at 3 h cold stress.tif]

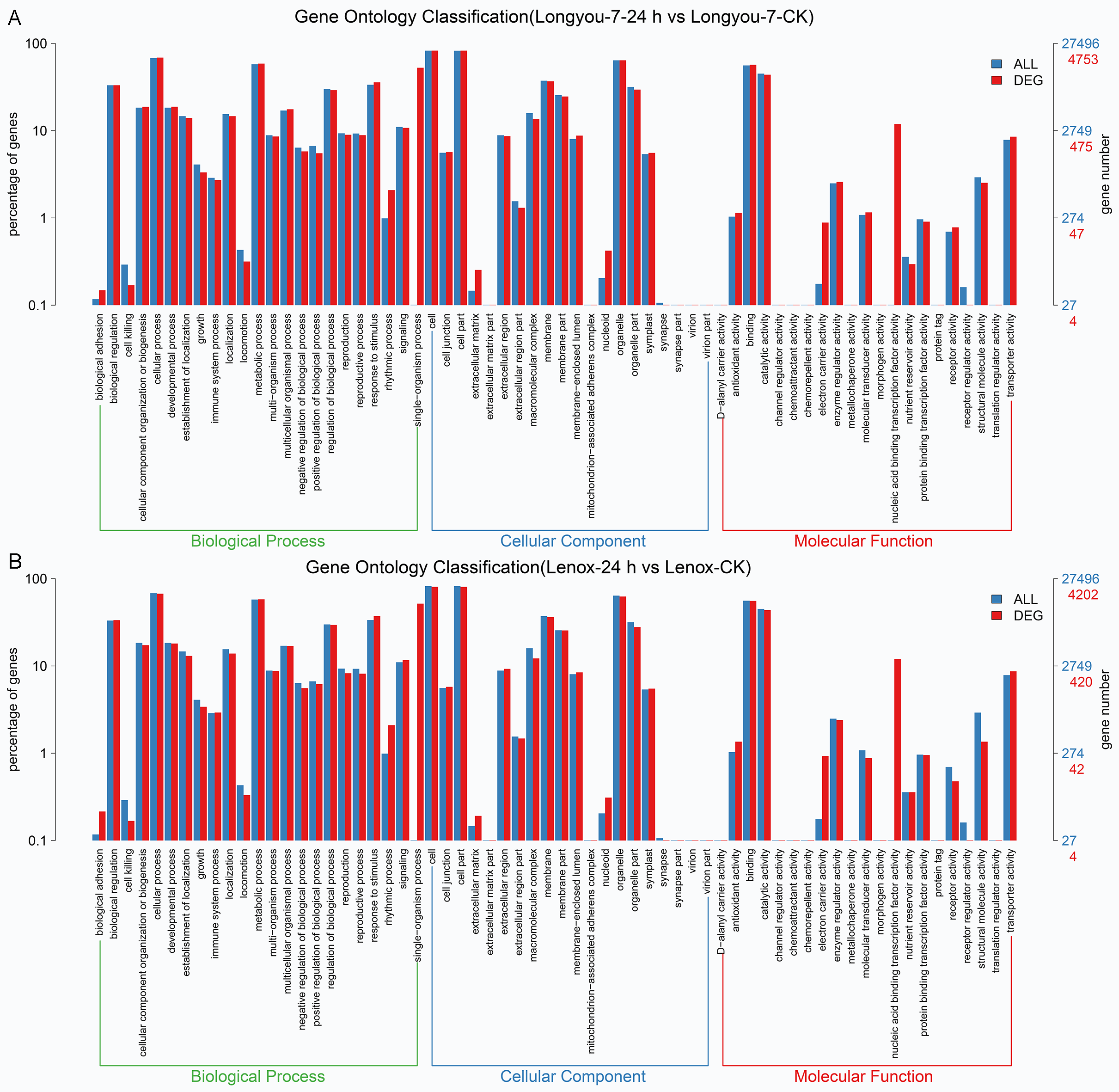

Supplement: Supplementary file 1 [file ijms-20-01071-s001.zip › Figure S5 GO Level 2 map of all expressed genes and DEGs at 24 h cold stress.tif]
